# Inborn Errors of Immunity in Latvia: Analysis of Data from 1994 to 2020

**DOI:** 10.1007/s10875-022-01229-1

**Published:** 2022-02-24

**Authors:** Tatjana Prokofjeva, Zane Lucane, Zanna Kovalova, Natalja Kurjane

**Affiliations:** 1 Children’s Clinical University Hospital, Vienibas gatve Street 45, Riga, LV-1004 Latvia; 2 Pauls Stradins Clinical University Hospital, Pilsonu Street 13, Riga, LV-1002 Latvia; 3 Riga Stradins University, Dzirciema Street 16, Riga, LV-1007 Latvia

To the Editor,

Inborn errors of immunity (IEI) refer to a heterogeneous group of congenital disorders that are characterised by impaired function and dysregulation of the immune system. IEI were traditionally considered to be rare disorders (1 in 10,000–1 in 50,000 live births), but the collective prevalence is now estimated to be around 1 in 1000–1 in 5000 [1]. Prevalence is an important epidemiological indicator that is used for planning public health policies. However, the prevalence of these conditions in Latvia is unknown.

To describe the epidemiological and clinical characteristics of patients with inborn error of immunity in Latvia over a 27-year period, we conducted a retrospective study, reviewing the medical records of patients who were diagnosed with an IEI in Latvia from the 1st of January 1994 to 31st of December 2020. Data were sourced from the only tertiary centres, where immunologists consult: The Children’s Clinical University Hospital and Pauls Stradins Clinical University Hospital (for adults) in Riga, Latvia, where severe patients from Latvia are treated.

Data regarding patients’ demographic and clinical characteristics were collected using the computerised hospital information systems and medical records. Diagnosis of IEI was based on the diagnostic criteria of the European Society for Immunodeficiencies (ESID). We retrospectively reviewed the data of these patients and re-evaluated the compliance with the ESID diagnostic criteria.

All data were analysed using Microsoft Office Excel and IBM SPSS Statistics for Windows Version 23. Data related to demographic and clinical indicators were analysed using descriptive statistics and parametric/non-parametric analysis, as appropriate. To estimate the minimum point prevalence and incidence, we used data sourced from the Central Statistical Bureau of Latvia in March 2021 [2]. The point prevalence was expressed as alive patients per 100,000 inhabitants at a particular point in time (December 2020). Incidence was presented as the number of patients per live births.

Over a 27-year period, a total of 173 patients with IEI were identified (see Table 1), with 47% being male (male/female ratio = 1:1.1). Patients with selective IgA deficiency (183 patients, median age 7.5 (IQR: 9.2), 59% of them male) and transient hypogammaglobulinaemia of infancy (4–7 patients per year) were not included in the study population. The median age of living patient in this cohort was 14.3 years (IQR: 31.3). Most of these patients (80%) were diagnosed before the age of 18 years. The median age at the time of diagnosis was 2.9 years (IQR: 15.9), and it ranged from birth to late adulthood (0–74 years). No statistical significance was found for age at diagnosis between male and female patients (*p* = 0.130). Thirty-nine (23%) patients died during the study period; the median age at death was 2.8 years (IQR: 14.4), ranging from 2 days to 51 years.

**Table 1:** Patients with Inborn errors of immunity in Latvia

	Number of patients	Gender M/F	Mortalities alive/deceased	Genetic testing yes/no/no information	Prophylactic antibiotics yes/no information	Immunoglobulin replacement therapy yes/no/no information	HSCT done/not done	Family history of IEI positive/negative/no information
Immunodeficiencies affecting cellular and humoral immunity	20 (12%)	12/8	2/18	8/12	0/20/0	14/6*/0	4/16	8/12/0
Combined immunodeficiencies with associated or syndromic features	57 (33%)	21/36	52/5	54/3	4/52/1	4/52/1	1/56	3/54/0
Predominantly antibody deficiency	36 (21%)	20/16	30/6	5/31	7/23/6	32/3/1	1/35	4/19/13
Diseases of immune dysregulation	8 (5%)	6/2	4/4	5/1/2	1/5/2	1/5/2	0/8	5/1/2
Congenital defects of phagocyte number or function	18 (10%)	12/6	18/0	10/2/6	6/2/10	0/8/10	4/14	5/3/10
Defects in intrinsic and innate immunity	2 (1%)	0/2	2/0	2/0	2/0/0	2/0/0	0/2	0/2/0
Auto-inflammatory disorders	8 (5%)	3/5	8/0	5/3	0/6/3	0/6/3	0/8	2/4/2
Complement deficiencies	11 (6%)	0/11	11/0	0/11	0/11/0	0/11/0	0/11	7/4/0
Bone marrow failure	11 (6%)	6/5	5/6	0/0/11	0/0/11	0/11/0	5/6	2/9/0
Unclassified inborn error of immunity	2 (1%)	1/1	2/0	1/1	1/0/1	0/1/1	0/2	0/1/1

^*^Patients who died prior to 2000 (except from 1994 to 1996) did not receive immunoglobulin replacement therapy, but plasma infusions instead

Genetic testing was performed in 52% of cases. Of these 173 patients, 31% of patients were recorded to have received IgG supplementation therapy, 10% received prophylactic antibacterial therapy, and 9% of patients received haematopoietic stem cell transplantation (HSCT), with a median age at time of HSCT of 2.1 years (IQR: 13.0). Familial cases were observed in 21% of patients.

Categories based on the International Union of Immunological Societies (IUIS) classification were distributed as shown in Table 1. The point prevalence of IEI in December 2020 in Latvia was 7.1 per 100,000 inhabitants (see Suppl. Fig. 1). The estimated 27-year incidence of severe combined immunodeficiency (SCID) in Latvia was 1 per 32,963 live births.

These data represent the first description of the epidemiology of IEI in Latvia. The prevalence of IEI in Latvia has increased tremendously, if compared to 0.08 per 100,000 in 1994 and 1.1 per 100,000 inhabitants in 2004, when Latvia joined the J project, to 7.1 per 100,000 inhabitants in 2020 (see Suppl. Fig. 1). The prevalence in other European countries has been reported between 1.3 per 100,000 inhabitants in Russia and 18.8 per 100,000 in Iceland [3, 4].

Predominantly antibody deficiencies composed 21% of cases; however, most studies regarding the epidemiology of IEI in other European countries have reported a larger proportion of patients with predominantly antibody deficiency: ranging from 20% in Iceland to 63% in Italy [4, 5]. A smaller proportion of patients diagnosed with predominantly antibody deficiency in combination to considerably stronger representation of paediatric patients could be due to the under-diagnosis of common variable immunodeficiencies. The rate of under-diagnosis could be high for several reasons: low availability of immunologic testing at the beginning of the study period, insufficient knowledge about IEI among non-immunologists, and low public awareness. We hypothesise that a certain number of patients with PFAPA could also be undiagnosed for this reason.

Awareness campaigns could be a solution to improve the public awareness of IEI in Latvia. In 2018, the Centre of the Rare Diseases was established in the Children Clinical University Hospital, and all IEI patients will be registered there; however, no national registries for IEI exist. Also, the availability and accessibility of immunologic testing should be improved.

The estimated incidence of SCID in Latvia was 1 per 32,963 live births. However, newborn screening for SCID is not available in Latvia; for this reason, patients with SCID that die in early infancy could be undiagnosed. The introduction of newborn screening could improve the care for these patients.

Regarding the diagnostics, genetic testing was performed in 52% of all IEI patients. Pathogenic variants were found in 18 genes (see Suppl. Table 1). The highest proportion of patients with genetically verified diagnoses was among the patients from the IUIS classification category combined immunodeficiencies with associated and syndromic features (54/57; 94.7%). Most genetically diagnosed patients had a Del 22q11.2 defect (35 patients). The diagnosis of 22q11.2 deletion was based on chromosome and fluorescence in situ hybridization (FISH) analysis. It was followed by five Familial Mediterranean fever patients with heterozygous (3) or compound heterozygous (2) *MEFV* gene mutation (three patients had Armanian; two—Slavic origin), and five patients with Nijmegen syndrome who all carried the typical Eastern Slavic mutation NM_002485.5(NBN):c.657_661del (p.Lys219fs); the origin of all five patients was Slavic. Five chronic granulomatous disease patients had a mutation in *CYBB* gene. Rare defects, with less than five patients for each gene, affected the following genes: *RAG2, IL2RG, DCLRE1C, CD40LG, WAS, ATM, SPINK5, KMT2D, KDM6A, PIK3CD, BTK, STAT1, PRF1, SH2D1A,* and *SBDS* (see Suppl. Table 1). The proportion of patients with a genetically confirmed diagnosis in Latvia has been increasing since 2018, when the public funding for the genetic testing abroad (Blueprint Genetics, Helsinki, Finland) became available. Before that, genetic testing was possible due to collaboration with the J project network.

Regarding the treatment, all patients who had indications for immunoglobulin replacement therapy (31% of all IEI patients) received IgG supplementation therapy. In comparison with reports from other countries, Latvia had the lowest proportion of patients receiving HSCT (9%); in other countries, this proportion ranged up to 16% in Russia [3]. This was due to limited health care resources in Latvia at the beginning of the study period. The possibility of sending patients abroad to receive HSCT has improved within the last few years.

This is, to the best of our knowledge, the first study examining the prevalence of IEI in Latvia. The main strength of the study is the long study period. Several limitations must also be considered. There were some limitations in the clinical data available in the medical records; therefore, a prospective collection of data would have been more accurate compared to our retrospective design. Another potential limitation is that some patients could be undiagnosed or not referred to immunologists, meaning that the prevalence could be underestimated.

## Supplementary Information

Below is the link to the electronic supplementary material.

Supplementary file1 (PDF 144 KB)

## Data Availability

The datasets generated during and/or analysed during the current study are available from the corresponding author on reasonable request.
