# Supplementary material for: Inborn Errors of Immunity in Latvia: Analysis of Data from 1994 to 2020
Source: J Clin Immunol. 2022 Feb 24;42(4):876–9. doi: 10.1007/s10875-022-01229-1 (PMC9166829; doi:10.1007/s10875-022-01229-1)
Supplement: Supplementary file 1 — Supplementary file1 (PDF 144 KB) [file 10875_2022_1229_MOESM1_ESM.pdf]

Tatjana Prokofjeva[1] and Zane Lucane[2,3], Zanna Kovalova[1], Natalja Kurjane[1,2,3]

Inborn errors of immunity in Latvia: Analysis of data from 1994 to 2020

[1]Children's Clinical University Hospital, Vienibas gatve Street 45, Riga, Latvia, LV-1004
[2]Paula Stradins Clinical University Hospital, Pilsonu Street 13, Riga, Latvia, LV-1002
[3]Rigas Stradins University, Dzirciema Street 16, Riga, Latvia, LV-1007
Corresponding author: Zane Lucane, email: zane.lucane@gmail.com; phone: +371-29289797, ORCID iD: 0000-0002-5356-2037

Supplementary Figure 1.

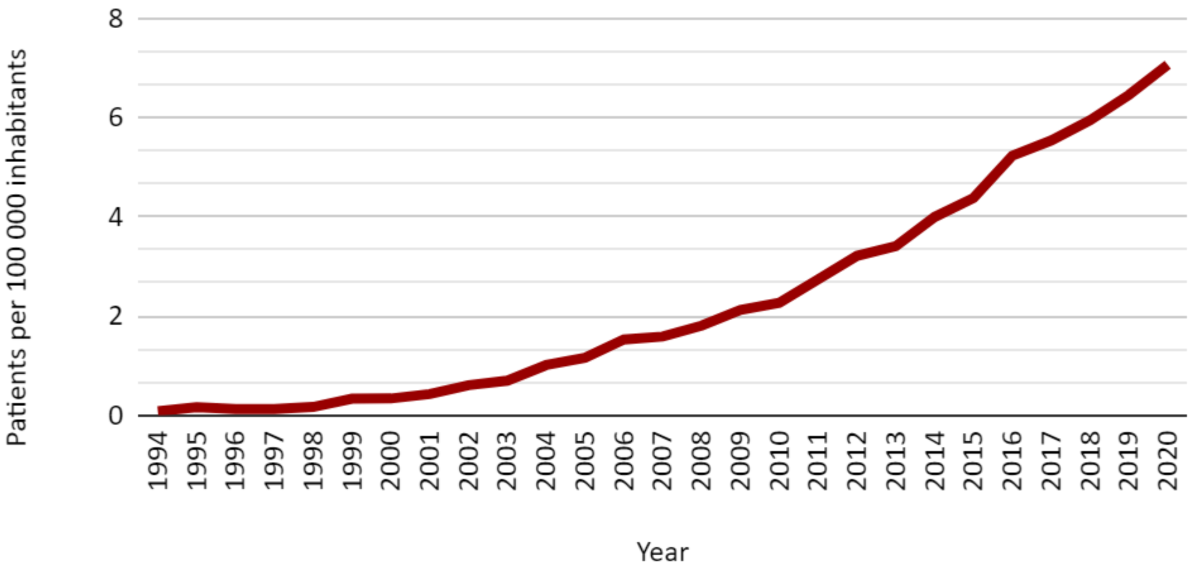

Supplementary Figure 1. Alive patients per 100 000 inhabitants in Latvia

Supplementary Table 1. Results of genetic testing in IEI patients in Latvia

**Immunodeficiencies affecting cellular and humoral immunity**
Severe combined immunodeficiency, undifferentiated phenotype<br>NM_000536.4(RAG2):c.595G>T (p.Glu199Ter) (1)
T-B+ severe combined immune deficiency<br>NM_000206.3(IL2RG):c.670C>T (p.Arg224Trp) (1)<br>Direct fluorescent sequencing of the *CD3Z* and *CD3G* genes did not show a mutation (1)
T-B- severe combined immune deficiency<br>Homozygous deletion DCLRE1C, EX1-3DEL,(?_-38)_(246-?)del (2)<br>*DCLRE1C* gene. Information about mutation is not available (1)
CD40 ligand deficiency

CD40L c.511C>A, (p.S184X) (2)
**Combined immunodeficiencies with associated or syndromic features**
Wiskott-Aldrich syndrome<br>NM_000377.3(WAS):c.631C>T (p.Arg211Ter) (1)
Ataxia-telangiectasia<br>Heterozygous NM_000051.4(ATM):c.1564_1565del (p.Glu522fs) (1)<br>NM_000051.4(ATM):c.5932G>T (p.Glu1978Ter); NM_000051.4(ATM):c.8731A>C (p.Thr2911Pro) (1)
Nijmegen breakage syndrome<br>NM_002485.5(NBN):c.657_661del (p.Lys219fs) (5)
22q11 deletion syndrome<br>22q11.2 deletion (based on FISH analysis) (35)
Comel-Netherton syndrome<br>NM_006846.4(SPINK5):c.1048C>T (p.Arg350*) (3)<br>Compound heterozygous NM_006846.4(SPINK5):c.1048C>T (p.Arg350*); c.1430+4A>G (1)
Kabuki syndrome<br>KMT2D c.3809C>G, p.(Ser1207*) (1)<br>KTM2D c.11377c>T, p.(Gln379*) (1)<br>KMT2D c.2728_2729dup, p.(Pro912Cysfs*) (1)<br>Duplication in Xp11.3 region (based on array comparative genomic hybridization data) (KDM6A gene) (1)<br>Information about mutations is not available (3)
**Predominantly antibody deficiency**
BTK deficiency, X-linked agammaglobulinaemia (XLA)<br>NM_000061.3(BTK):c.1082A>G (p.Tyr361Cys) (1)<br>BTK c.391+1G>A, IVS5+1G>A (1)
Common variable immune deficiency with no gene defect specified (CVID)<br>PID panel inconclusive (1)
Activated p110δ syndrome (APDS)<br>NM_005026.5(PIK3CD):c.1573G>A (p.Glu525Lys) (1)<br>Heterozygous NM_005026.3(PIK3CD):c.2905C>T, p.(Arg969Cys) (1)
**Diseases of immune dysregulation**
Familial haemophagocytic lymphohistiocytosis, Perforin deficiency 2 –<br>Compound heterozygous NM_001083116.3(PRF1):c.658G>A (p.Gly220Ser);<br>NM_001083116.3(PRF1):c.666C>A (p.His222Gln) (2)
X-linked lymphoproliferative disease (XLP1)<br>[GRCh37]Xq25(123479999_123672135)x0 (SH2D1A gene) (2)<br>NM_002351.5(SH2D1A):c.5A>G (p.Asp2Gly) (1)
**Congenital defects of phagocyte number or function**

Shwachman-Diamond Syndrome<br>NM_016038.4(SBDS):c.258+2T>C; c.183-184TA>CT (1)
X-linked chronic granulomatous disease<br>NM_000397.4(CYBB):c.676C>T (p.Arg226Ter) (3)<br>CYBB c.1166G>T, (p.Gly389Val) (1)<br>CYBB c.577T>C, p.(Ser193Pro) (1)
Congenital neutropenia<br>Information about mutations is not available
**Defects in intrinsic and innate immunity**
NM_007315.4(STAT1):c.812A>C (p.Gln271Pro) (1)<br>NM_007315.4(STAT1):c.1154C>T (p.Thr385Met) (1)
**Auto-inflammatory disorders**
Familial Mediterranean fever<br>Heterozygous NM_000243.3(MEFV):c.2080A>G (p.Met694Val) (1)<br>Heterozygous NM_000243.3(MEFV):c.2177T>C (p.Val726Ala) (1)<br>Heterozygous NM_000243.3(MEFV):c.442G>C (p.Glu148Gln) (1)<br>Compound heterozygous NM_000243.3(MEFV):c.2080A>G (p.Met694Val); NM_000243.3(MEFV):c.2177T>C (p.Val726Ala) (2)
**Unclassified inborn error of immunity**<br>PID panel inconclusive (1)
